# External Glands of *Nepenthes* Traps: Structure and Potential Function

**DOI:** 10.3390/ijms26167788

**Published:** 2025-08-12

**Authors:** Bartosz J. Płachno, Małgorzata Kapusta, Marcin Feldo, Piotr Stolarczyk, Karol Małota, Krzysztof Banaś

**Affiliations:** 1 Department of Plant Cytology and Embryology, Institute of Botany, Faculty of Biology, Jagiellonian University, 9 Gronostajowa St., 30-387 Kraków, Poland; 2 Bioimaging Laboratory, Faculty of Biology, University of Gdańsk, 59 Wita Stwosza St., 80-308 Gdańsk, Poland; malgorzata.kapusta@ug.edu.pl; 3 Department of Vascular Surgery and Angiology, Medical University of Lublin, 16 Staszica St., 20-081 Lublin, Poland; martinf@interia.pl; 4 Department of Botany, Physiology and Plant Protection, Faculty of Biotechnology and Horticulture, University of Agriculture in Kraków, 29 Listopada 54 Ave., 31-425 Kraków, Poland; piotr.stolarczyk@urk.edu.pl; 5 Institute of Biology, Biotechnology and Environmental Protection, Faculty of Natural Sciences, University of Silesia in Katowice, 9 Bankowa St., 40-007 Katowice, Poland; karol.malota@us.edu.pl; 6 Department of Plant Ecology, Faculty of Biology, University of Gdańsk, 59 Wita Stwosza St., 80-308 Gdańsk, Poland; krzysztof.banas@biol.ug.edu.pl

**Keywords:** pitcher plants, carnivorous plants, cell wall, cell wall microdomains, trichomes, hydathodes, hemicelluloses, glands, scanning transmission electron microscopy, pectic homogalacturonan, xyloglucan, xylan

## Abstract

*Nepenthes* L. species (tropical pitcher plants) are a classic example of carnivorous plants. The *Nepenthes* traps are highly specialized pitchers with a zoned structure. On the outer surface of the pitcher, there are nectaries and various types of trichomes, including glandular trichomes. The main aim of our study was to examine these glandular trichome structures and check the distribution of the homogalacturonans (HGs) and hemicelluloses in the cell wall of trichome cells. The structure of *Nepenthes bicalcarata* Hook. f. and *Nepenthes albomarginata* T.Lobb ex Lindl. trichomes was analyzed using light and electron microscopy. The antibodies were used against the wall components [anti-pectic homogalacturonans (HGs): JIM5 (low methylesterified HGs), LM19 (low methylesterified HGs), CCRC-M38 (a fully de-esterified HGs), JIM7 (highly esterified HGs), LM20 (esterified HGs), LM5 (galactan) and anti-hemicelluloses: LM25 (xyloglucan), LM15 (galactoxyloglucan), CCRC-M138 (xylan), and LM10 antibody (xylan)]. The localization of the examined compounds was determined using immunohistochemistry techniques. The presence of endodermal and transfer cells supports the idea that peltate trichomes actively transport solutes. Also, the presence of pectic homogalacturonans and hydrophilic hemicelluloses indicates that water or aqueous solutions are transported through the trichomes’ cell walls. Our study supports the idea that these trichomes may act as hydathodes or hydropotes.

## 1. Introduction

**Figure 1:**
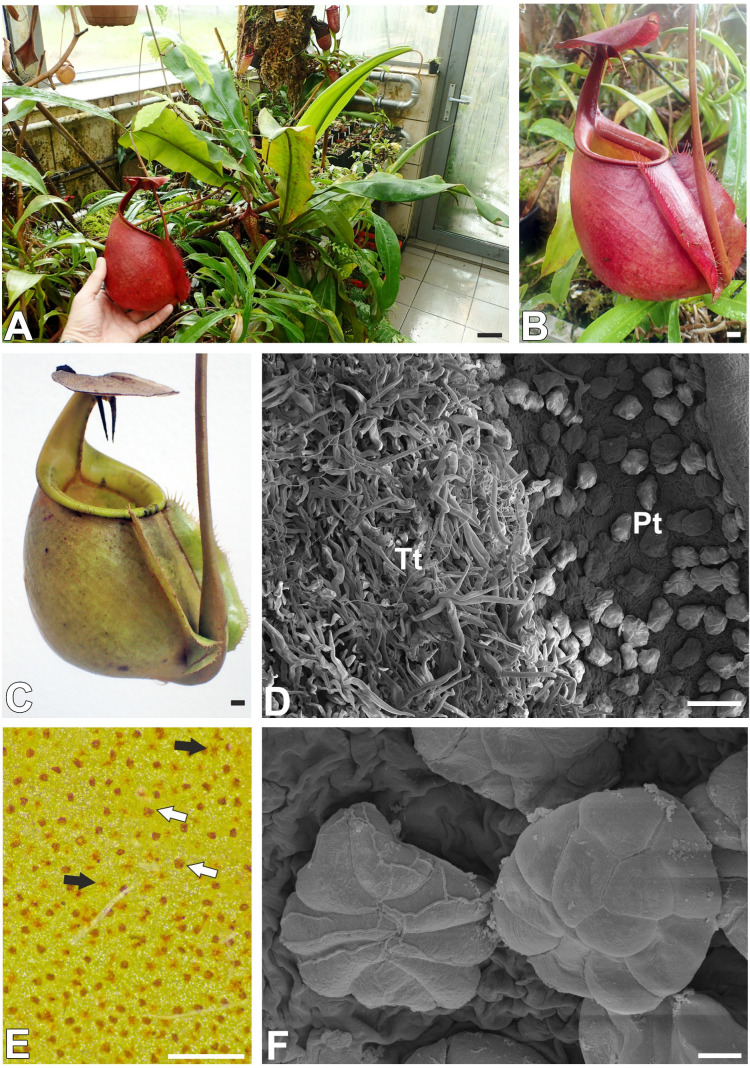
Plant and trap morphology of *Nepenthes bicalcarata* Hook. f. (**A**) A cultivated plant, bar = 4 cm. (**B**,**C**) The morphology of the pitchers, bar = 1 cm. (**D**) An accumulation of peltate trichomes (Pts) and tufted trichomes (Tts) near the peristome, (scanning electron microscopy—SEM), bar = 100 µm. (**E**) Outer surface of the pitcher, featuring visible trichomes of both types: peltate trichomes (white arrow) and tufted trichomes (black arrow), bar = 500 µm. (**F**) Morphology of peltate trichomes (scanning electron microscopy—SEM), bar = 10 µm.

Carnivorous plants (commonly known as insectivorous plants) are mixotrophic organisms that perform photosynthesis but derive some compounds (nutrients; mainly N, P, K, and Mg) from other organisms using specialized traps, which are mainly transformed and specialized leaves. This allows these plants to exist in nutrient-poor habitats [1,2,3,4,5]. The genus *Nepenthes* L. (tropical pitcher plants) is a classic example of such plants. These plants were discovered as early as the 17th century [6], and later in the Victorian era, they were popular as exotic plant oddities for the aristocracy. This genus (monotypic Nepenthaceae, Caryophyllales [7,8]) includes vines or subscandent shrubs. This genus is restricted to the Palaeotropics and has the center of diversity in Southeast Asia [9,10]. After *Utricularia* spp. [11], it is the largest genus of carnivorous plants [10], comprising about 200 species, and numerous new *Nepenthes* species are being discovered today [12,13,14,15,16]. All *Nepenthes* species produce traps in the form of pitchers. The *Nepenthes* leaf consists of an extremely enlarged leaf base and a tendril that carries the pitcher. Most species have two types of pitchers: lower (terrestrial) and upper (aerial). At first, the young plant produces a rosette of leaves with the lower pitchers and, after reaching an appropriate age and growth, produces pitchers of the second type. These types differ in shape and some of their structural characteristics [6,17,18], and the spectrum of captured prey. Generally, upper pitchers specialize in capturing flying insects [18,19]. The pitcher can be divided into zones related to attraction, retention of prey, digestion of prey bodies, and absorption of nutrients [1,6]. These zones are characterized by different morphology and the presence of specialized structures. Juniper et al. [1] distinguished four pitcher zones. The lid and peristome with associated nectaries comprise the first “attractive zone”. The second zone is a “conductive, slippery zone”, and here, the inner pitcher wall is wax-coated. According to Juniper et al. [1], the third zone is functionless. Benz et al. [20] treated it as a transitional zone between the slippery and digestive zones. The last zone is called “absorptive” or “digestive” and is equipped with digestive glands. Specialized pitcher surfaces related to carnivory have aroused the interest of scientists from various fields; these surfaces include the peristome, e.g., [21,22,23,24,25,26], wax layer [20,21,27,28,29,30,31,32,33], and digestive glands [21,34,35,36,37,38,39,40,41]. This is partly related to the development of new materials based on the characteristics of biological surfaces and advances in biomechanics, e.g., [42,43,44,45,46]. For example, *Nepenthes* peristome inspired the creation of a new material called “SLIPS”—Slippery Liquid-Infused Porous Surface [47,48]. However, the outer surface of *Nepenthes* spp. pitchers attracted less attention. Fenner [49] showed various trichomes (hairs) from the outer surface of the pitchers of *Nepenthes rafflesiana* Jack. Lloyd [6] classified them into two groups: tufted trichomes and sessile stellate trichomes. Macfarlane [50] pointed out that stellate trichomes can absorb water. According to Stern [51], who also described the development of stellate trichomes, these stellate trichomes function as hydathodes. Also, Metcalfe and Chalk [52] noted them as hydathodes. Interestingly, Gorb and Gorb [32] proposed that these trichomes secrete a sugar-containing water solution. The detailed structure and histochemistry of these glandular trichomes have not been sufficiently understood. For example, it is not known whether they contain an endodermal component, which would confirm their glandular functions. Thus, the main aim of the study was to study glandular trichome structure and also check the distribution of the pectic homogalacturonans (HGs) and hemicelluloses in the cell wall of trichome cells. Analysis of cell walls, both in terms of their composition (the presence of hydrophobic compounds that inhibit apoplastic transport and hydrophilic compounds such as pectic homogalacturonans and hemicelluloses, which facilitate transport) and structure (e.g., the presence of cell wall ingrowths), can help us to understand the functioning of trichomes and their role. In particular, such data are available when it comes to trichomes from the outer surfaces of traps in *Dionaea muscipula* J.Ellis [53] and *Aldrovanda vesiculosa* L. [54]. As the main model for the study, we chose *Nepenthes bicalcarata* Hook. f. (Figure 1A–C). Additionally, we used *Nepenthes albomarginata* T.Lobb ex Lindl.

## 2. Results

### 2.1. Trichome Structure (Nepenthes bicalcarata)

**Figure 2:**
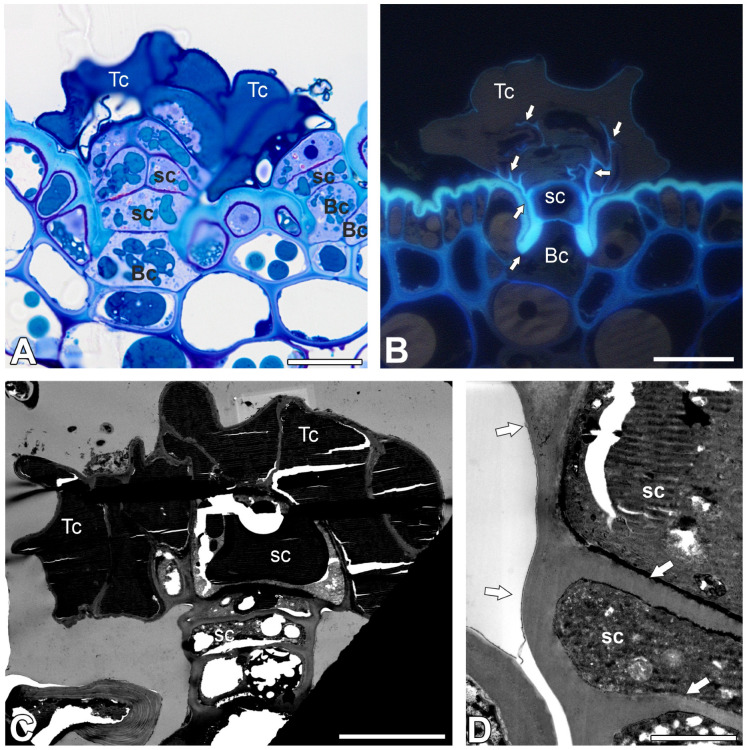
Structure of the peltate trichomes of the *Nepenthes bicalcarata* pitcher: Hc—head cell; sc—stalk cell; Bc—basal cell. (**A**) A semi-thin section of two peltate trichomes, bar = 20 µm. (**B**) Section of a peltate trichome; autofluorescence of the cell walls. Note the strong autofluorescence of the cutinized cell walls of the stalk cells, terminal cells, and basal cells (arrow), bar = 25 µm. (**C**) Ultrastructure of peltate trichome (scanning transmission electron microscopy—STEM), bar = 10 µm (**D**) Ultrastructure of a stalk cell; note the cutinized cell wall of the stalk cells (arrow); (transmission scanning electron microscopy—STEM), bar = 1 µm.

On the outer surface of the pitcher (trap) there were two types of trichomes. The first group consisted of glandular (peltate) trichomes, and the second group consisted of tufted trichomes. An accumulation of peltate trichomes in the form of a ring occurred near the peristome (Figure 1D). Below, there is an accumulation of tufted trichomes Figure 1D), between which there were also peltate trichomes. Both types of trichomes are distributed on the outer part of the pitcher (Figure 1E). Peltate trichomes were flattened (Figure 1F). Each trichome was situated in small, shallow depression. Each trichome consisted of a single large basal cell or two smaller cells (this component had contact with epidermal and parenchyma cells), a stalk, and a head (Figure 2A–C).

The distal part of the lateral–outer cell wall of the basal cell was modified and highly cutinized (Figure 2B). In contrast, the proximal lateral part of the cell wall was not cutinized. Plasmodesmata connected basal cells to the parenchyma cells. These plasmodesmata occurred in pits. The basal cell was connected by plasmodesmata to the stalk cell.

**Figure 3:**
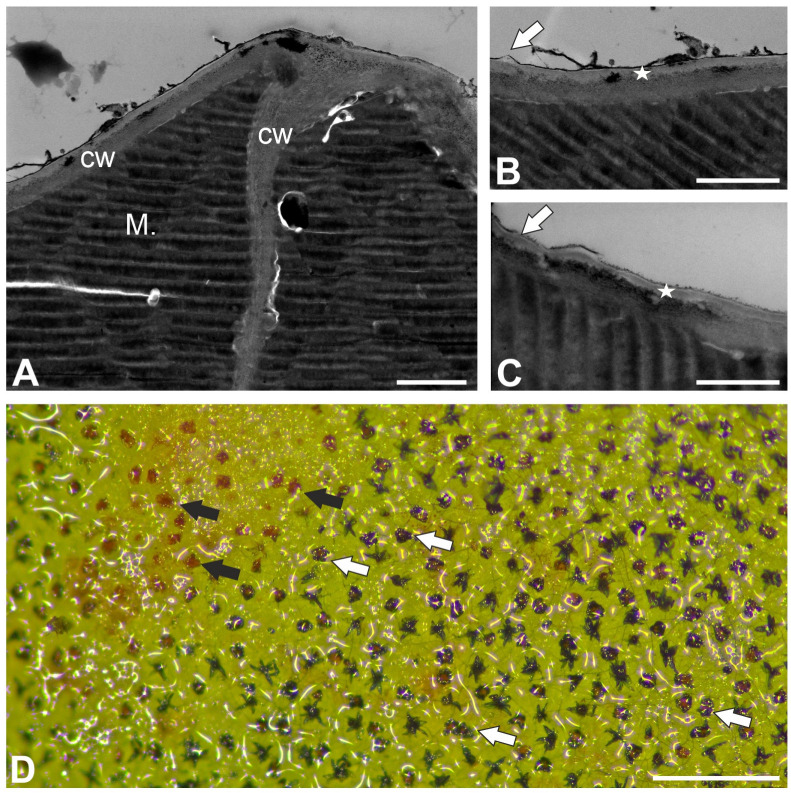
Structure of the peltate trichomes of the *Nepenthes bicalcarata* pitcher. (**A**) Ultrastructure of terminal cells, material in vacuole (M), cell wall (cw), bar = 1 µm. (**B**,**C**) Details the cuticle structure of terminal cells; discontinuous in cuticle (arrow), cutinized cell wall (star), bar = 500 nm. (**D**) Pitcher epidermis with peltate trichomes (white arrows), treated with toluidine blue; the glands have absorbed the dye. Trichomes that have not been in contact with the dye are brown (black arrow), bar = 500 µm.

The stalk was multicellular (up to seven cells), the lower part was uniseriate, and the upper part was biseriate. The outer cell walls of stalk cells were cutinized (Figure 2B–D). The anticlinal cell walls between stalk cells were also cutinized in some parts (Figure 2B–D). The contents of the vacuoles stained strongly with methylene blue/azure II (Figure 2A). This material is electron-dense, and as such was poorly permeated with resin, which tended to fall out (Figure 2C). The head was multicellular; the number of cells depended on the trichome (up to 12 terminal cells). Depending on the trichome, the terminal cells may or may not have collapsed. Terminal cells were almost entirely filled with vacuoles containing material that showed characteristic corrugations (visible on sections by both light and electron microscopy) (Figure 2A,C and Figure 3A).

The contents of the vacuoles were successfully stained with methylene blue/azure II (Figure 2A), but the staining was less intense than that of the vacuole contents of stalk cells. The contents of the vacuoles did not show positive staining after the periodic acid–Schiff (PAS) reaction. The cell nucleus was located in the basal part of the cell, along with a small amount of cytoplasm containing mitochondria and endoplasmic reticulum. Cell walls between terminal cells had small swells. External cell wall of terminal cells had a well-developed cutinized part which formed a layer (Figure 3B,C). There were discontinuous in cuticle (Figure 3B,C). After application of toluidine blue, both tufted and peltate trichomes were stained (Figure 3D).

### 2.2. Pectic Homogalacturonan and Galactan Distribution

**Figure 4:**
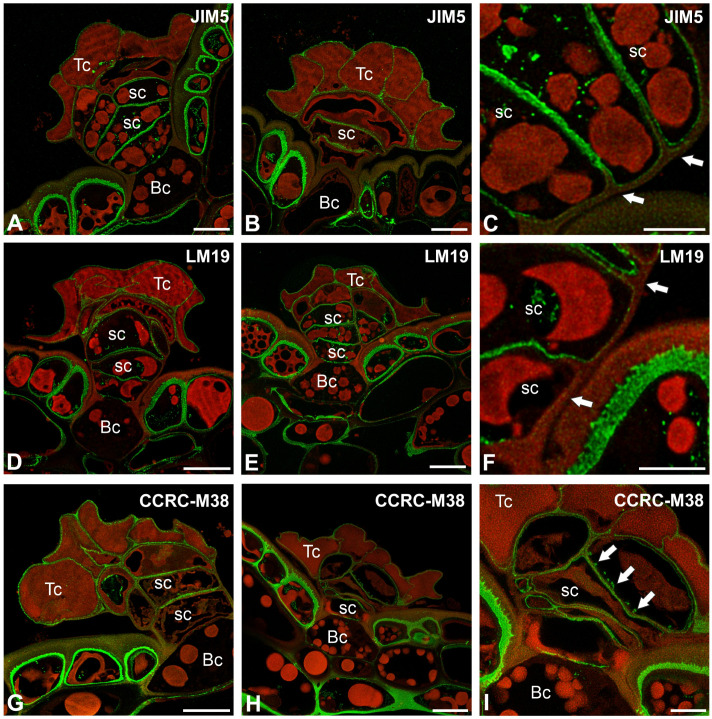
Pectic homogalacturonan distribution in the peltate trichomes of *Nepenthes bicalcarata* pitcher (intense green color—antibody signal; red-brown color—autofluorescence); Hc—head cell; sc—stalk cell; Bc—basal cell. (**A**,**B**) Labeling of cells with JIM5 (low methylesterified HG) in the peltate trichomes, bar = 10 µm. (**C**) An enlargement of figure A showing no positive signal at wall modification sites (arrow), bar = 5 µm. (**D**,**E**) Labeling of cells with LM19 (low methylesterified HG) in the peltate trichomes, bar = 10 µm. (**F**) An enlargement of figure D showing no positive signal at wall modification sites (arrow), bar = 5 µm. (**G**,**H**) Labeling of cells with CCRC-M38 (low methylesterified HG) in the peltate trichomes, bar = 10 µm. (**I**) An enlargement of figure H showing positive signal in cell wall ingrowths (arrow), bar = 5 µm.

The epitope recognized by the JIM5 antibody (low methylesterified HGs) was detected in the cell walls of terminal and stalk cells (Figure 4A–C). The signal was absent in the thick cell walls of basal cells, which were impregnated with cutin. Also in stalk cells, the signal was absent in these parts of the cell walls, which were impregnated with cutin (Figure 4C). A fluorescence signal detected by JIM5 (low methylesterified HGs) was observed in the inner part of the outer cell walls of epidermal cells. The epitope recognized by LM19 antibody (low methylesterified HGs) had a similar distribution to the epitope recognized by the JIM5 antibody (Figure 4D–F). The fluorescence signal detected by CCRC-M38 (a fully de-esterified HGs) was observed in the cell walls of terminal cells, stalk, and basal cells (Figure 4G–I). The signal was absent in the thick cell walls of basal cell, which were impregnated with cutin. Also in stalk cells, the signal was absent in these parts of the cell walls which were impregnated with cutin (Figure 4G–I). The positive signal occurred in the cell wall ingrowths in the stalk cells (Figure 4I).

**Figure 5:**
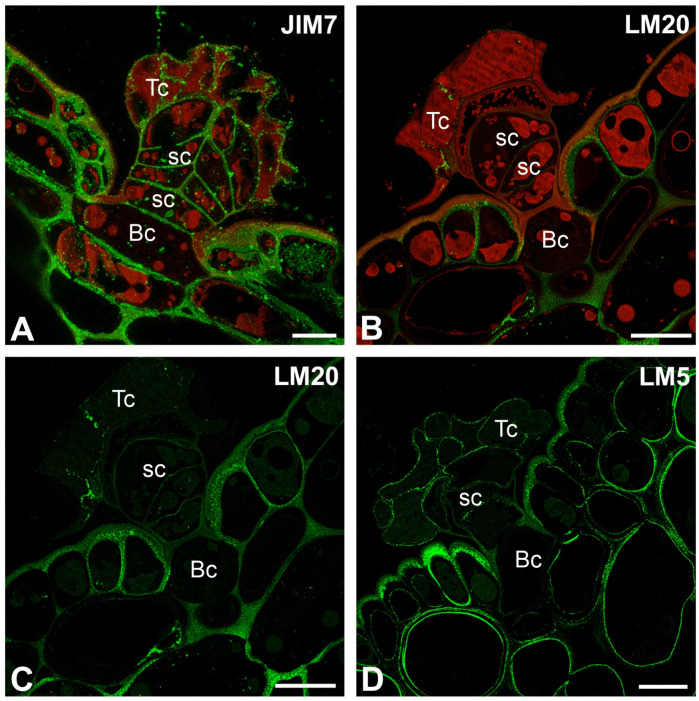
Pectic homogalacturonan and galactan distribution in the peltate trichomes of *Nepenthes bicalcarata* pitcher (intense green color—signal of antibody, red-brown color—autofluorescence); Hc—head cell; sc—stalk cell; Bc—basal cell. (**A**) Labeling of cells with JIM7 (methylesterified HG) in the peltate trichomes, bar = 10 µm. (**B**,**C**) Labeling of cells with LM20 (methylesterified HG) in the peltate trichomes, bar = 10 µm. (**D**) Labeling of cells with LM5 (galactan) in the peltate trichomes, bar = 10 µm.

A fluorescence signal from highly esterified HGs (detected by JIM7) was observed in the cell walls of terminal cells, stalk, and basal cells (Figure 5A). The signal was absent in the thick cell walls of basal cell, which were impregnated with cutin; however, the signal was in the most outer and inner parts of these cell walls. There was also no signal in the side cell wall of the stalk cell, where impregnation occurred (Figure 5A).

A delicate fluorescence signal from esterified HGs (detected by LM20) was observed in stalk cells; however, there was also no signal in the side cell wall of the stalk cell, where impregnation occurred (Figure 5B,C).

The signal from the pectic polysaccharide (1–4)-β-D-galactan (detected by LM5) was observed in the cell walls of terminal cells, and stalk cells (Figure 5D).

### 2.3. Hemicellulose Distribution

**Figure 6:**
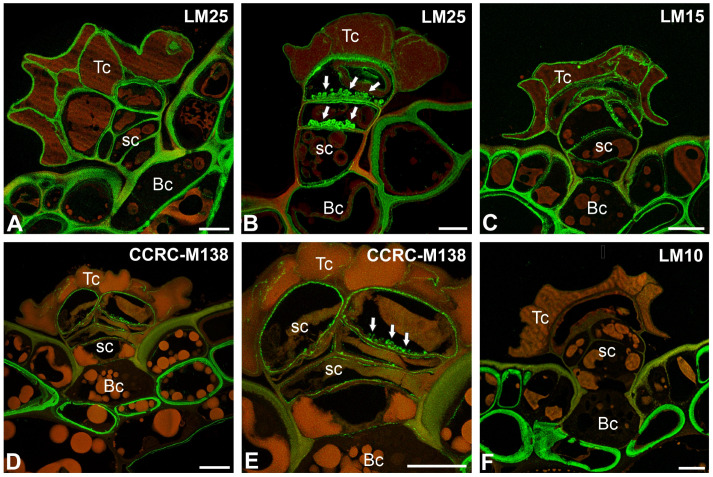
Hemicellulose distribution in the peltate trichomes of *Nepenthes bicalcarata* and *Nepenthes albomarginata* pitcher (intense green color—signal of antibody, red-brown color—autofluorescence); Hc—head cell; sc—stalk cell; Bc—basal cell. (**A**) Labeling of cells with LM25 (galactoxyloglucans) in the peltate trichomes of *Nepenthes bicalcarata,* bar = 10 µm. (**B**) Labeling of cells with LM25 (galactoxyloglucans) in the peltate trichomes of *Nepenthes albomarginata*; note the cell wall ingrowths (arrow), bar = 5 µm. (**C**) Labeling of cells with LM15 (xyloglucan) in the peltate trichomes of *Nepenthes bicalcarata*, bar = 10 µm. (**D**) Labeling of cells with CCRC-M138 (xylan) (xyloglucan) in the peltate trichomes of *Nepenthes bicalcarata*, bar = 10 µm. (**E**) An enlargement of figure D showing positive signal in cell wall ingrowths (arrow), bar = 10 µm. (**F**) Labeling of cells with LM10 (xylan) in the peltate trichomes of *Nepenthes bicalcarata*, bar = 5 µm.

The epitope recognized by the LM25 antibody (which recognizes land plants’ galactoxyloglucans) was detected in the cell walls of epidermal and hypodermal cells (Figure 6A), as well as in the cell walls of trichome cells (Figure 6A). In *Nepenthes albomarginata*, a positive signal was observed in the wall ingrowths of stalk cells (Figure 6B). The epitope recognized by the LM15 antibody (which reacts with the land plants’ xyloglucans) was detected in the cell walls of epidermal and hypodermal cells (Figure 6C). The strong fluorescence signal of the antibody occurred in the cell walls of basal, stalk, and head cells (Figure 6C). The signal was also observed in modified cell walls.

The strong fluorescence signal detected by CCRC-M138 (which recognizes the glycan group of Xylan-6) was observed in the cell walls of hypodermal cells (Figure 6D). Epidermal cells differed in terms of signal presence. If a signal was present, it was in the inner part of the cell wall, whereas there was no signal in the walls impregnated with cutin. The fluorescence signal detected by CCRC-M138 was also detected in the cell walls of basal, stalk, and head cells (Figure 6E); however, the signal was absent in these parts of the cell walls, which were impregnated with cutin (Figure 6D). A positive signal was observed in wall ingrowths of stalk cells (Figure 6E).

The epitope recognized by the LM10 antibody (which recognizes land plant xylan) was absent in cell walls of trichome cells (Figure 6F).

### 2.4. Histochemistry Staining (Dye Staining)

**Figure 7:**
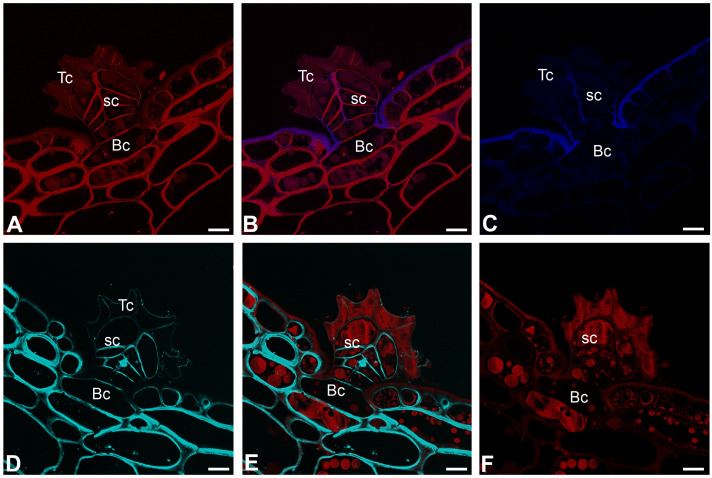
Dye staining the peltate trichomes of *Nepenthes bicalcarata*; Hc—head cell; sc—stalk cell; Bc—basal cell. (**A**–**C**) A section of the trichome stained by Carbotrace 680 (red color); cutin-impregnated cell wall fluorescence (blue color). (**D**–**F**) A section of the trichome stained with Calcofluor White (blue color) and propidium iodide (red color). All bars = 5 µm.

The cell walls of trichome cells were intensively stained with Carbotrace 680, except for cutin-impregnated cell walls (Figure 7A–C). Similarly, the cell walls of trichome cells were intensively stained with Calcofluor White, except for a cutin-impregnated region (Figure 7D–F).

### 2.5. The Cell Viability Test

**Figure 8:**
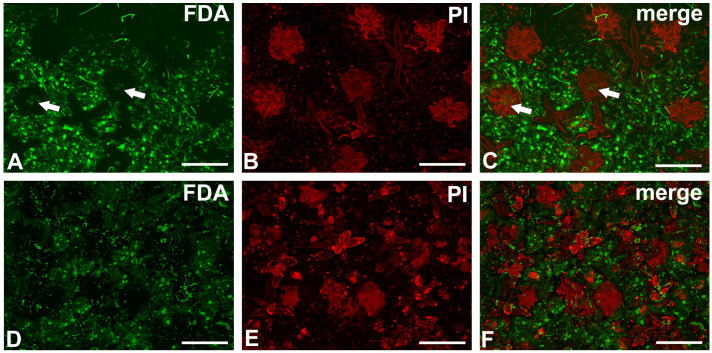
The trichome cell viability test of the peltate trichomes of *Nepenthes bicalcarata*. (**A**–**C**) Trichome head cell viability test in a young but opened pitcher. A fluorescein signal is visible in the central part of the trichomes (arrow); also note the fungal hyphae. (**D**–**F**) Trichome head cell viability test in an older pitcher. Note that the trichomes were stained with only propidium iodide without a fluorescein signal; all bars = 100 μm.

In young but opened pitchers, signal of fluoresceine diacetate was seen in the central part of some trichomes (Figure 8A–C). Most of the head cells of the peltate trichomes were dead in older pitchers (Figure 8D–F). However, a fluorescein signal was visible in stomata cells.

## 3. Discussion

Trichomes in plants are very differently structured and have different functions [55,56]. One of the functions of trichomes is to create defense mechanisms against parasites and herbivores. This can be a physical barrier or may provide insect and herbivore deterrence or pest immobilization [56,57,58,59,60]. Young *Nepenthes* pitchers are densely covered by both tufted and glandular trichomes [61]. Since young organs are the most vulnerable to pest damage in many plant species, trichomes and other glandular structures protect them. In the case of *Nepenthes bicalcarata*, such protection may be insufficient because young pitchers can be destroyed by weevils (*Alcidodes* sp.) if ants do not protect them [62]. Trichomes are not an obstacle for insects, which move easily on the outer surface of the pitcher. We did not observe secretions such as mucilage or resin on the surface of the peltate trichomes (mature pitcher), so the external surface of the pitcher was not sticky. Trichomes, therefore, are not a physical barrier to pests (at least to insects).

Stern [51] and Metcalfe and Chalk [52] proposed that peltate trichomes may participate in the absorption or release of water; however, they did not provide any arguments to support this hypothesis. According to Fahn [63], glandular trichomes should have complete cutinization of the side walls of stalk cells, similar to that occurring in the walls of the cells of the root endodermis (it should be noted that endodermis and its Casparian strips occur not only in roots, but also in angiosperm shoots [64]). The presence of such endodermal cells (with the Casparian strip) causes a blockage of apoplastic transport. Therefore, transport must occur via the symplastic route, thanks to which the plant controls the transport. Our studies show that peltate trichomes contain endodermal cells, which confirms that these trichomes have a glandular function.

Interestingly, there are several endodermal cells in one *Nepenthes* spp. trichome, especially as we compare these trichomes with the external glandular trap trichomes of the Lentibulariaceae Rich. [65,66,67,68], nectary trichomes of Lentibulariaceae [69,70,71] or the trap trichomes in the Byblidaceae Domin [72], in which there is only one endodermal cell per trichome. In trichomes from the outer surfaces of traps in *Dionaea muscipula* J.Ellis [53], there are four endodermal cells per trichome. *Aldrovanda vesiculosa* L. bifid trichomes [54] have two endodermal cells per trichome. In large glands, e.g., the digestive gland of *Nepenthes* spp. [61] and glandular emergences of *Drosera* spp. [73,74], *Drosophyllum lusitanicum* (L.) Link [75], and *Triphyophyllum peltatum* (Hutch. & Dalziel) Airy Shaw [76], there are many endodermal cells, which form an endodermoid layer [1]. The arrangement of endodermal cells in a row in the *Nepenthes* trichome may also play a role in reinforcing the structure of the trichome, especially since the side walls of the basal cell of the trichome are highly cutinized.

The second argument for the involvement of peltate trichomes in transport is the presence of transfer cells in the stalk. Transfer cells have cell wall ingrowths that increase the surface area of the plasma membrane, facilitating efficient solute exchange across apoplast/symplast boundaries [77,78,79]. Here, we found cell wall ingrowths in trichomes of both *Nepenthes* studied. Our observations confirm the previous results of cell wall ingrowths in *Nepenthes* based on the PAS method [80]. Transfer cells have been identified in abaxial/external trap surface trichomes of other carnivorous plants: *Utricularia* [66,67,81], *Drosera* [1,82], *Dionaea muscipula* [53], and *Aldrovanda vesiculosa* [54].

The next argument for the involvement of *Nepenthes* peltate trichomes in transport is the presence of cuticular discontinuities (permeable cuticles) that allow transport (absorption or secretion). Using an aqueous solution of toluidine blue, we have confirmed that trichomes can absorb.

All these observations support the ideas of Stern [51] and Metcalfe and Chalk [52], who have stated that the peltate trichomes transport solutes and may act as hydathodes or hydropotes. Although the terminal cells die, the stem cells remain alive for some time and can perform an active transport function. Terminal cells form a large apoplastic space, which could be used for the transportation of solutions similar to external trap trichomes of *Dionaea muscipula*.

However, the suggestion by Gorb and Gorb [32] that peltate trichomes secrete a sugar-containing water solution is incorrect, because glands acting as nectaries on the outer surface of the pitcher are present glands that have a different structure. Also, we did not observe sugar secretion on the surface of trichomes (mature pitchers).

We observed osmiophilic material in the vacuoles in the trichome cells; a similar material was observed in the gland cells, which synthesize anthocyanins and naphthoquinones [83]. It is known that *Nepenthes* cells produce naphthoquinones, which are used for protection against herbivory [84,85,86,87]. These chemicals are also produced in the pitcher tissues [88]. Therefore, we cannot rule out that these trichomes may be involved in producing naphthoquinones; however, proving this requires further analysis.

We found that demethyl-esterified pectic HG epitopes in *Nepenthes* occurred in the cell walls of trichome cells. This is in contrast to bifid trichomes and digestive glands from *Aldrovanda vesiculosa* traps [54,89,90] and in stellate trichomes in *Dionaea muscipula* [53], where the cell walls of terminal cells were poor in low-esterified HGs. Both methylesterified and demethylesterified pectic HG epitopes were abundant in the cell walls of quadrifid cells (in basal, pedestal, and terminal cells) of *Utricularia* [90]. Demethylesterified pectic HG epitopes in *Nepenthes* were especially abundant in the cell walls of stalk cells. The demethylesterified pectic HG epitopes were absent where the cell wall was modified (cutinization). This was also the case for epidermal cells, where the outer cell wall was cutinized, while epitopes were present in the inner layer of the cell wall, which was not cutinized. We found that methylesterified pectic HG epitopes detected by LM20 in *Nepenthes* were not abundant in the cell walls of trichome cells, in contrast to the epitopes recognized by the JIM7 antibody, which were present. These differences in occurrence demonstrate that different groups of pectins may have other roles or properties. Pectic HGs are involved in plant cell wall porosity and hydration [91,92]; thus, the occurrence of these pectic HGs in the cell walls of peltate trichome cells is important for transporting substances through the cell walls. In the cell walls of peltate trichome cells, we found various hemicelluloses (xylan, galactoxyloglucan, and xyloglucan). Also, the presence of these hydrophilic hemicelluloses indicates that water or aqueous solution transport occurs through the trichomes’ cell walls.

Carbotrace 680 labels cellulose [93,94,95] and hemicellulose xyloglucan [96]. In the case of peltate *Nepenthes* trichomes, staining results obtained using Carbotrace 680 agreed with staining results obtained for Calcofluor White Stain. For both dyes, negative staining occurred in cell walls impregnated with cutin. The same negative staining result was in epidermal cells in the outer part of the cell walls, which were impregnated with cutin. We have previously shown that Carbotrace 680 is unsuitable for analyzing wall components in walls saturated with hydrophobic substances (cutin) in *Utricularia* glands. Now we see that this is repeated in unrelated species. The occurrence of hemicelluloses partially overlaps with Carbotrace 680 staining. However, there were some differences, e.g., the hemicelluloses recognized by the LM15 antibodies (for xyloglucan) were also present in cell walls that were impregnated with cutin.

It should be noted that *Nepenthes bicalcarata* is a noteworthy species due to its unique nectar secreting structures (the two giant thorn-like nectaries) [62], the weak digestive power of the pitcher’s fluid [29], the reduction in the wax zone [17], and mutualism with ants *Camponotus schmitzi* [97,98,99,100]. Also, *Nepenthes albomarginata* is specific due to its specialization in capturing termites [101].

Because *Nepenthes* are an extremely diverse group with different strategies for obtaining nutrient compounds and with various specializations in the structure and function of traps, e.g., [46,102,103,104], in the future, it will be interesting to study the structure of peltate trichomes in different species.

## 4. Materials and Methods

### 4.1. Plant Material

*Nepenthes bicalcarata* Hook. f. plants were grown in the warm greenhouse of the Faculty of Biology, University of Gdansk. *Nepenthes albomarginata* T.Lobb ex Lindl. pitchers were taken from the collection of the first author.

### 4.2. Histological and Immunochemical Analysis

The pitchers were cut into small fragments and fixed as described by Płachno et al. [90]. For analysis of the occurrence of the major cell wall polysaccharides and glycoproteins, the plant material was dehydrated with acetone and embedded in an Epoxy Embedding Medium Kit (Fluka). Ultrathin sections were cut on a Leica Ultracut UCT ultramicrotome. The rehydrated sections in PBS buffer were blocked with 1% bovine serum albumin (BSA, Sigma-Aldrich) in a PBS buffer and incubated with the following primary antibodies overnight at 4 °C: anti-homogalacturonans (HGs): JIM5 (low methylesterified HGs), JIM7 (highly esterified HGs), LM19 (low methylesterified HGs), CCRC-M38 (fully de-esterified HGs), LM20 (esterified HG), LM5 (galactan) and anti-hemicelluloses: LM25 (xyloglucan), LM15 (galactoxyloglucan), CCRC-M138 (xylan), LM 10 (xylan) [105,106,107,108,109,110,111,112,113]. All of the primary antibodies were used in a 1:20 dilution. They were purchased from Plant Probes, UK (rat monoclonal antibodies: JIM5, JIM7, LM19, LM25, and LM15), and from Agrisera, Sweden (mouse monoclonal antibodies: CCRC-M38, CCRC-M1, and CCRC-M138). Secondary antibodies—goat anti-rat secondary or anti-mouse antibody conjugated with FITC or Alexa Fluor 488, respectively—were purchased from Abcam (Cambridge, UK). The samples were then cover-slipped using a Mowiol mounting medium: a mixture of Mowiol ^®^4–88 (Sigma-Aldrich) and glycerol for fluorescence microscopy (Merck, Warsaw, Poland) with the addition of 2.5% DABCO (Carl Roth GmbH + Co. KG, Karlsruhe, Germany). They were viewed using a Leica STELLARIS 5 WLL confocal microscope with lightning deconvolution. At least two replications were performed for each of the analyzed traps, and about five to ten sections from each organ were analyzed for each antibody used. Negative controls were created by omitting the primary antibody step, which caused no fluorescence signal in any of the control frames for any stained slides (Figure S1). Semi-thin sections (0.7–1.0 µm thick) were prepared for light microscopy (LM) and stained for general histology using aqueous methylene blue/azure II (MB/AII) for 1–2 min.

Cellulose and hemicellulose xyloglucan were labeled using Carbotrace 680 (Ebba Biotech AB, Nobels väg 16 S-171 65 Solna, Sweden; https://www.ebbabiotech.com/products/carbotrace-680?variant=47885141180748 (accessed on 4 December 2024)). Crystalline cellulose was also labeled using Calcofluor White Stain (Merck Life Science Sp.z.o.o., an affiliate of Merck KGaA, Darmstadt, Germany). Sections were viewed using a Leica DM6000B microscope equipped with a DAPI (Ex/Em = 350/450 nm wavelength; exposure time, 347.136 ms with gain = 1) and a Rhodamine filter (Ex/Em = 546/585 nm wavelength; exposure time, 661.156 ms with gain = 1.9).

Toluidine blue solution was applied to the pitcher’s surface to see if trichomes can take up aqueous solutions. After several hours, the pitcher was rinsed with water and photos were taken.

### 4.3. Scanning Transmission Electron Microscopy (STEM)

The glands were also examined using electron microscopy, as follows: Fragments of the traps were fixed in a mixture of 2.5% glutaraldehyde with 2.5% formaldehyde in a 0.05 M cacodylate buffer (Sigma-Aldrich Sp. z o.o., Poznań, Poland; pH 7.2) for a few days, and later, the material was processed as in the work of Płachno et al. [114]. The material was dehydrated with acetone and embedded in an Epoxy Embedding Medium Kit (Fluka) or Durcupan resin (Sigma-Aldrich Chemie GmbH, Taufkirchen, Germany). Ultrathin sections were cut on a Leica Ultracut UCT ultramicrotome. The sections were examined using a Hitachi UHR FE-SEM SU 8010 microscope housed at the University of Silesia in Katowice.

### 4.4. Scanning Electron Microscopy

For the scanning electron microscopy (SEM), the traps were fixed in a mixture of 2.5% glutaraldehyde with 2.5% formaldehyde in a 0.05 M cacodylate buffer and later washed in buffer, transferred to ethanol, and then transferred to acetone and dried using supercritical CO_2_. A part of the material was fixed in 100% methanol, transferred to ethanol, and then transferred to acetone and dried using supercritical CO_2_. The material was then sputter-coated with gold and examined using a Hitachi S-4700 scanning electron microscope (Tokyo, Japan), which is housed at the Institute of Geological Sciences, Jagiellonian University, Kraków, Poland, or a Hitachi UHR FE-SEM SU 8010 microscope, which is housed at the University of Silesia in Katowice.

### 4.5. Head Cell Viability Test

Fragments of the pitchers of *Nepenthes bicalcarata* (young and older) with trichomes were immediately stained with a dual FDA/PI working solution. A fluorescein diacetate (FDA; Sigma-Aldrich Sp. z o.o., Poznań, Poland) stock concentration of 1 g/mL in acetone and 2 μg/mL working solution of PI (Sigma-Aldrich Sp. z o.o., Poznań, Poland) in a PBS buffer was used.

## 5. Conclusions

Our research confirms that *Nepenthes* peltate trichomes have glandular functions. This is evidenced, among other things, by endodermal cells (with Casparian strips), transfer cells (with cell wall ingrowths), and the discontinuous cuticles of terminal cells. The presence of pectic homogalacturonans and hydrophilic hemicelluloses indicates that transport of water or aqueous solutions may occur through the cell walls of trichomes. Terminal cells form large apoplastic space, which could be used to transport solutions. All these observations support the idea presented by Stern, and also Metcalfe and Chalk, that the peltate trichomes transport solutes and may act as hydathodes or hydropotes. Various species of *Nepenthes* show specialization in pitcher development and prey capture. It is therefore interesting to study more species of this genus in the future with regard to their external trichomes. It remains an open question whether, for example, *Nepenthes* species found in drier climates differ from those in wetter conditions in terms of the persistence and function of trichomes.

## Data Availability

The data presented in this study are available on request from the corresponding author.

## Supplementary Materials

The following supporting information can be downloaded at https://www.mdpi.com/article/10.3390/ijms26167788/s1.
