# Supplementary material for: External Glands of Nepenthes Traps: Structure and Potential Function
Source: Int J Mol Sci. 2025 Aug 12;26(16):7788. doi: 10.3390/ijms26167788 (PMC12386587; doi:10.3390/ijms26167788)

Figure S1

Figure S1. Control reactions of cell wall components after immunolabeling (green color – signal of antibody), (**A**-**B**). Section through the peltate trichome: (**A**) An autofluorescence signal collected for the FITC channel, (**B**) An autofluorescence signal collected for the Alexa 488, both bars = 10 um.

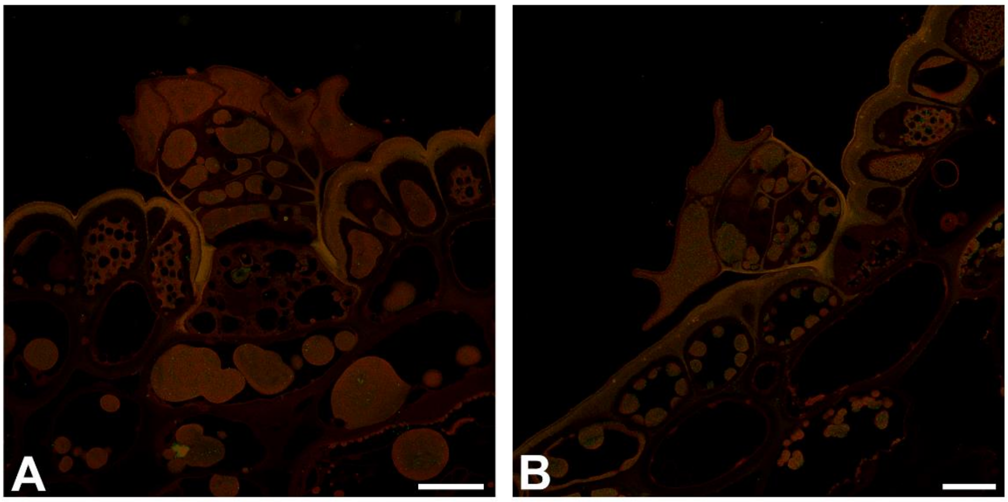

Supplement: Supplementary file 1 [file ijms-26-07788-s001.zip › ijms-3757187-supplementary.pdf]
